# Sex Differences in Autism Spectrum Disorders: Does Sex Moderate the Pathway from Clinical Symptoms to Adaptive Behavior?

**DOI:** 10.1038/srep10418

**Published:** 2015-05-19

**Authors:** Vanja Mandic-Maravic, Milica Pejovic-Milovancevic, Marija Mitkovic-Voncina, Milutin Kostic, Olivera Aleksic-Hil, Jelena Radosavljev-Kircanski, Teodora Mincic, Dusica Lecic-Tosevski

**Affiliations:** 1 Institute of Mental Health, Palmoticeva 37, Belgrade, Serbia; 2 School of Medicine, University of Belgrade, Serbia; 3 Faculty of Media and Communication, University of Singidunum, Belgrade, Serbia; 4 Serbian Academy of Sciences and Arts, Belgrade, Serbia

## Abstract

We explored sex differences in diagnostic categories, clinical symptoms and adaptive behavior of persons with autism spectrum disorders, as well as sex-specific correlations of clinical and adaptive caracteristics. The study involved 108 patients (83 males, 6.73 ± 4.33 years old) diagnosed with autism spectrum disorders (ASD). Assessment included ADI-R and Vineland Adaptive Behavior Scale II. Males were more often diagnosed with typical autism. There were no sex differences in the autistic symptoms, while females showed better functioning in Daily living skills, without reaching statistically significant difference (p = 0.062). We have found different associations of autistic symptoms with different aspects of adaptive behavior in males and females. Social reciprocity in females correlated with social domain of adaptive behavior, in a positive direction. Our findings have shown that although there are no sex differences in autistic symptoms, females tend to be somewhat more functional, and are also less frequently diagnosed with typical autism. Our results have also shown that sex might moderate the way clinical symptoms are expressed in adaptive behavior. Social reciprocity might be the core feature regarding sex differences in ASD. Our findings might have diagnostic and therapeutical implications, pointing out to the need for individualized, sex-specific treatment in this group of disorders.

Autism is a disabling developmental disorder estimated to be present in every 88^th^ child and every 54^th^ boy in the USA, according to the USA Center for Disease Control data, with the latest 2008 analyses showing the 23% increase in autism prevalence since 2006^1^. Autism spectrum disorders (ASD) are a group of conditions characterized by three cardinal clinical features: qualitative impairment in social interactions and impaired verbal and non-verbal communication, as well as a restricted range of interests. ASD are also characterized by early onset, delay of development of key psychological functions, change of symptomatic expression by age and a chronic course with various levels of disability^2^. The classification is under revision – the DSM-5 contains and ICD-11 is expected to contain only two domains: (1) social communication/social interaction and (2) restricted, repetitive behaviors and interests^3^. It has been suggested that the concept of a spectrum of autism as a whole is more appropriate in clinical work than the existing subgroups^4^, which emphasizes the importance of dimensional assessment of autistic disorders^5^. Still, in the current ICD-10 classification, ASD include defined clinical entities^6^.

Studies based on clinical and epidemiological samples have suggested a higher incidence of autism in boys than in girls, with ratios reported averaging around 3-4:1^2^,^7^,^8^. The specific male-to-female ratio in ASD diagnoses, as a firm and replicated finding, has been explained by two approaches. The first approach explains this finding through differences in biology of male and female brain - there is a difference in brain structure between males and females, the amygdala is larger in boys than in girls, and there are more neurons in male cerebral cortex^9^. Another hypothesis postulated by Baron-Cohen introduces the “extreme male brain”, emphasizing the difference between male and female cognitive and affective styles. Males are oriented towards systemizing, while females towards understanding and empathizing^9^. This approach attempts to answer the question whether males are biologically more prone to ASD, but does not explore specific differences in ASD manifestation between sexes and the possibility of more frequent *diagnostic detection* in males. The male-to-female ratio differences reported in the studies may actually result from the underestimated number of females with ASD due to their “non-male-typical” presentation, leading to underdiagnosed ASD in females^10^. A recent study has shown that girls are less likely to meet diagnostic criteria for ASD at equivalently high levels of autistic-like traits and one of the suggested explanations of the finding was possibly better adaptation/compensation in girls^11^.

In order to define whether there are sex differences in presentation of ASD once it *is* diagnosed (the second approach), a number of studies explored the ASD phenotype in males and females. So far, the findings have not been consistent. Several studies on this issue found less repetitive, restrictive and stereotyped behavior (RRSB) in female than in male patients^12^,^13^. In one study with a higher functioning sample of adults with ASD, only females with developmental language delay had lower current performance IQ^10^. A study done on toddlers with ASD showed that girls with ASD evidenced greater communication deficits than boys, and boys evidenced more restricted, repetitive, and stereotyped behavior than girls^14^. On the other hand, some studies found no differences for the triad of autism core dysfunctions^10^,^15^,^16^.

In addition to inconsistent findings in different studies regarding clinical differences in ASD between sexes, there is insufficient data to make conclusions about sex differences in level of adaptive behavior in ASD. Adaptive behavior is the capacity to accomplish conceptual, social and practical demands on a daily basis^17^. In ASD, adaptive behavior may be as, if not more, useful as a measure of outcome than assessments of cognitive abilities^18^. In ASD, difficulties in adaptive behavior might be life-long, they are very important for prognosis, and also implicate large social and economic burden^19^. Examining both clinical manifestations and functional impairment, as well as their interaction in male vs. female patients with ASD, might be one of important steps towards understanding biological mechanisms that underlie this group of disorders^20^. Therefore, our study was designed to assess sex differences, not only in clinical symptoms, but also in the way the disorder reflects itself in everyday life, by exploring adaptive behavior. Furthermore, this study was aimed to explore how clinical symptoms and adaptive behavior are interconnected in each sex, hypothesising that it might be the sex itself that moderates how autistic symptoms are expressed.

## Method

The study was performed during 2009-2013 at a university psychiatric clinic.

### Participants

The study involved 108 patients (83 males, 25 females, 6.73 ± 4.33 years old), diagnosed with ASD, recruited as consecutive referrals. Prior to participation, parents or caregivers of all participants signed the informed consent.

The inclusion criterion was the presence of any of the ASD. The diagnosis was verified by the clinical criteria of the ICD-10^6^ confirmed by a referring child psychiatrist trained in diagnosing autism. It was done through clinical interview with a parent and examination of a child. Besides clinical criteria, the diagnosis was verified by the Autism Diagnostic Interview – Revised (ADI-R)^21^, conducted by a trained child psychiatrist.

### Instruments

*Autism Diagnostic Interview – Revised (ADI-R*^*21*^). ADI-R is a standardized semistructured parent/caregiver interview, designed for assessment of signs of autism and ASD. It consists of 93 items, and explores child’s early development, aquisition of language skills, language and communication functioning, loss of both speech and motor skills, social development and play, interests and behavior. The parent/caregiver description for each item is given for childhood (ever) and current behavior. Specific items regarding social reciprocity, communication and RRSB are used to calculate the scores in these three domains (ADI-R A, ADI-R B and ADI-R C score, respectively). Higher scores account for worse symptoms. The interview was administered by certified child psychiatrists. In our study, we used the *diagnostic algorithm* to determine whether autism criteria are met. In order to determine sex differences in the clinical manifestations of autism, we used the scores for *current* symptom severity. Also, to diminish the effect of language levels on algorithm scores^22^, we included only non-verbal children.

*Vineland Adaptive Behavior Scale, Second Edition (Vineland-II)*^23^ was used for the assessment of everyday adaptive behavior. The questions were answered by parents or caregivers and were related to description of patients adaptive behavior. The instrument assesses adaptive behavior in domains of Communication (C - receptive, expressive, and written communication skills), Daily Living Skills (DLS - personal behavior as well as domestic and community interaction skills), Socialization (S - play and leisure time, interpersonal relationships, and various coping skills) and Motor Skills (MS - gross and fine), providing the composite score (Vineland-II score) which summarises patients’ skills in all four domains. It was administered by certified child psychologists.

### Statistical analysis

We used descriptive statistics parameters (mean, standard deviation, frequencies) to present the data, t-test for independent samples and Chi^2^-test to analyze sex differences. Univariate analysis of covariance – ANCOVA was used to test sex differences, excluding the effects of age, adaptive behavior and ADI-R scores. Pearson’s correlations were used to assess sex-specific associations between age, ADI-R scores and adaptive behavior – both composite and domain scores. We used a series of linear regressions to explore predictive effects of age and ADI-R scores on adaptive behavior within each sex group. Finally, sex moderation analyses were done by using PROCESS (macro for SPSS)^24^, based on ordinary least square regression within path analytical framework.

## Results

In order to analyze sex differences in diagnoses, we assigned participants to two groups – those with typical form of autism - “childhood autism”, and those with other forms of ASD (any of other Pervasive Developmental Disorders (PDDs) according to ICD-10)^6^. Our findings have shown that males were more frequently diagnosed with typical forms of autism (83%) compared to females (56%) (Table 1a).

There were no sex differences in ADI-R current scores, without and with controlling for age (Table 1a). Analyzing the differences in adaptive behavior, we found higher scores in females for Vineland C and Vineland DLS, but without reaching a statistically significant difference (p = 0.062) (Table 1b).

In bivariate correlation analyses among males, age was significantly inversely related to all of the Vineland scores, with correlation coefficients ranging from -0.354 (p = 0.007) for MS score, to –0.779 (p = 0.000) for S score. Also, age was significantly inversely related to ADI-R A (r = –0.251; p = 0.022) and B scores (r = −0.545; p = 0.000), while it showed positive correlation with ADI-R C score (r = 0.483; p = 0.000). Among males, ADI-R B score was significantly related to Vineland Composite (r = 0.347; p = 0.001), DLS (r = 0.271; p = 0.013) and S (r = 0.284; p = 0.009) in a positive way, whereas ADI-R C score had significant inverse correlations with Vineland Composite (r = –0.493; p = 0.000), C (r = −0.414; p = 0.000), DLS (r = −0.524; p = 0.000), and S scores (r = –0.569; p = 0.000), and marginally significant inverse correlation with MS score (r = –0.245; p = 0.066). ADI-R A score was significantly related only to Vineland MS score in an inverse direction (r = –0.331; p = 0.012).

In females, age was significantly inversely associated with all Vineland-II scores, with correlation coefficients ranging from -0.612 (p = 0.005) for MS score, to -0.801 (p = 0.000) for Vineland Composite, while there were no significant associations between age and ADI-R scores. Also, ADI-R C score marginally significantly correlated with Vineland C score, in a negative direction (r = −0.377; p = 0.063).

In further analyses, we conducted sex specific linear regression models of each Vineland score, with age and ADI-R scores as predictors (Table 2). In males, Vineland scores were significantly predicted by the regression models, accounting for 63.3% of the Vineland Composite variance, 39.3% of the Vineland C variance, 61.4% of the Vineland DLS variance, 68.1% of the Vineland S variance, and 24.5% of the Vineland MS variance. Age was a significant predictor of all Vineland scores among boys, with negative direction of relationship. ADI-R A score was a significant predictor for Vineland Composite, DLS and MS scores, also in a negative direction. ADI-R C score was also a significant negative predictor for Vineland DLS and S scores. ADI-R B score was not a significant predictor for any of the Vineland scores in males.

In females, Vineland scores were also significantly predicted by the regression models, apart from the MS score (due to small number of females with MS score – it was not calculated for persons over 5 years of age). Significant prediction models accounted for 66.7% of the Vineland Composite variance, 58.5% of the Vineland C variance, 59.5% of the Vineland DLS variance, and 67.7% of the Vineland S variance. Among girls, age was a significant predictor of all Vineland scores (even for MS score which was not significantly predicted by the model), in a negative direction. ADI-R C score was a significant predictor for Vineland S score, and marginally significant (p = 0.059) for Vineland C score, both in a negative direction. ADI-R A score was a marginally significant predictor for Vineland S score (p = 0.063), but in a positive direction. As in males, ADI-R B score was not found to be a significant predictor for any of the Vineland scores in females.

Finally, we examined moderating effects of sex on relationship of each ADI-R score with Vineland scores, controlling for other ADI-R scores and age as covariates. We found significant moderation effects of sex on ADI-R A score relationship to Vineland DLS (with ADI-R A being significant negative predictor of Vineland DLS only in males) and to Vineland S score (with ADI-R A being significant positive predictor of Vineland DLS in females only) (Table 3).

## Discussion

In accordance with the theory that females are more often presented with “atypical autism” comparing to males^10^, our findings have shown that males were more often diagnosed with typical autism. Atypical presentation may be one of the causes of lower autism prevalence in females. In other words, it is possible that, especially in high functioning groups, autism is under-diagnosed in females^25^.

For the current clinical scores measured by ADI-R (social reciprocity, communication, RRSB), we found no differences between males and females. Several studies reported findings similar to ours, showing no sex differences in ADI-R scores^15^,^26^,^27^. Different findings from those of our study were shown in two recent studies with high functioning children and adults with autism^10^,^12^ which showed less RRSB in females. The reason for these differences might be a different sample – these studies were done on a high functioning sample, while our sample was low functioning. It might suggest that only high functioning girls show less repetitive interest than boys. A study done in children with ASD showed that females had more communication symptoms, while males had more RRSB symptoms^14^. This study, on the other hand, was carried out on a sample of toddlers, while our study included a wider age range.

When it comes to the level of adaptive behavior in our study, we found that girls had better score on Vineland DLS, but without statistically significant difference. Our female sample was small, therefore the differences could possibly reach statistical significance in a larger sample. There is no much data on sex differences in the literature. One study that explored sex differences in high and low risk groups for autism showed that females scored better on S and DLS scales^28^, which partly corresponds to our results.

In accordance with a recent study performed by Dworsynski and colleagues^11^, we suppose that girls might be less likely to meet diagnostic criteria for typical ASD (and probably any ASD diagnosis) at equivalent levels of autistic symptoms. The explanations offered by the aforementioned authors refer to a possibly better adaptation/compensation in girls. In our study, this may be reflected by the fact that both instruments were parent-administered, allowing for a girl with a given level of symptoms to be perceived by the parent as better functioning than a boy with a similar symptoms severity.

Several studies have shown that Vineland scores have a marked decrease with age in an autistic population^29^,^30^. It is also known for ADI-R to vary in the degree to which it is correlated with chronological age^31^. Our study examined sex-specific age-dependence of clinical symptoms and adaptive behavior, as well. Overall adaptive behavior was worse with higher age in both sexes, which is in accordance with previous studies^29^,^30^. Even when partialling out the effects of clinical symptoms in linear regression analyses, age was still a significant negative predictor for all of the Vineland scores in both sexes. However, when it comes to clinical symptoms, sex specific associations with age were found. While males had a pattern of better social reciprocity and communication, as well as of worse RRSB with older age, in females we found no age association with any of the ADI-R scores. One explanation of such results may be related to the smaller sample of female participants. On the other hand, this finding might imply a lack of symptom progress in girls through time, which is contrary to the results of a recent study among adults with ASD^10^ showing that females may achieve more progress in compensatory socio-communication ability (measured by Autism Diagnostic Observation Schedule **–** ADOS^31^).

Clinical symptoms are not necessarily fully and directly “translated” into functional outcomes, leaving some space for other potential factors to redeem/alleviate functional impairment^32^,^33^. The main goal of our study was to explore whether sex could be one of such factors. To our knowledge, studies that examined associations between autistic symptoms and adaptive behavior have not taken the influence of sex into consideration^32^,^34^, while some of them included only males^29^. Our findings might be in favor of our hypothesis that sex may affect the translation of symptoms into adaptive functioning. Sex-specific linear regressions revealed somewhat different symptom predictor profiles of adaptive behavior. Symptoms were predictive of the overall adaptive behavior in boys, but not in girls and they predicted more adaptive behavior domains in boys than in girls. When it comes to specific symptom predictors of adaptive behavior, sex differences were also found. Whereas worse social reciprocity predicted worse functioning in males (worse global functioning, daily living skills and motor skills), it predicted marginally better functioning in females (better socialization).

As the ultimate test to answer the main question of this study, the moderation analyses further confirmed the influence of sex on pathway from clinical symptoms to adaptive behavior, revealing the underlying importance of social reciprocity as its core. In case of worse social reciprocity, being male was a risk factor for the worse functioning in domain of daily living skills, whereas being female was a factor related to better functioning in domain of socialization. In a recent study, daily living skills have been found to be weaker in males not only in samples of children with ASD, but also in non-ASD high risk samples, as well as in non-ASD low-risk samples^28^. Our finding might therefore mirror the sex differences in general. The unexpected finding that being a female with worse social reciprocity symptoms leads to better social functioning could be also explained through general sex differences. A recent study even suggested gender-specific items for scales used in screening of autism spectrum disorders, with emphasis on differences in social-communicative difficulties^35^,^36^. “Normal boys” tend to play in groups which makes it difficult for them to join in, in contrast to “normal girls” who tend to play with one or two other girls^35^,^36^. Also, “normal girls” might be more sensitive and more frequently invite girls with developmental problems to join them^35^. If we analyze the items of Vineland S subscale, it might be that low social reciprocity in girls might leads to, for example, to better pretend play and better use of leisure time. This finding also might be a consequence of the better adaptation possibilities in autistic females, as argued by Dworzynski *et al.*^11^. It is also important to note that this finding was prominent for the feature considered to be “a typically female strength” – social reciprocity and functioning. Even if so, showing the moderation effect in both sexes regarding social reciprocity might point out that this clinical feature should be explored in more detail when it comes to sex differences in ASD.

In conclusion, our results have shown a different translational pattern of clinical symptoms into adaptive behavior between males and females with ASD, with males having a more predictable effect of clinical symptoms on adaptive behavior and everyday living skills, females having more predictable effect on socialization and communication domain, with social reciprocity being a core clinical feature affected by sex in terms of functional results, and with females being paradoxically perceived as less socialized when they are actually more socially interactive.

Since recent studies support the idea that focusing the treatment on adaptive behavior and not solely on clinical symptoms is a new priority in treatment of ASD^33^, the treatment might be oriented towards different aspects of adaptive behavior, with focus mostly on socialization and communication in girls, and a broader spectrum of adaptive behavior domains in boys, keeping in mind sex specific clinical risk factors. Different patterns shown in our study point out to a different approach in diagnostic as well as treatment planning in males and females with ASD.

Our study has certain limitations. The sample included consecutive referrals (convenience sample). This might have led to the fact that our sample was lower-functioning, with an assumption that parents of higher-functioning children are less prone to seek help of a specialist. Still, the male-to-female ratio in our sample was 4:1, and corresponds to the findings in general epidemiological studies^2^,^7^,^8^. The age of participants in our study was not homogenous, but it was controlled for in the statistical analyses. Furthermore, although the number of females in our sample (N = 25) is similar to ranges in previous studies - from 23 to 52^10^,^12^,^15^, it still might be considered small. Further limitation of our study might be that all data were obtained from parent-reported measures. Still, a recent review study of diagnostic tools for ASD determined that ADI-R, along with ADOS stands out with the highest sensitivity and specificity^37^.

### Ethical standards

This study has been approved by the local ethics committee and has been performed in accordance with the principles of good clinical practice. Prior to participation in this study, parents/caretakers signed the informed consent.

## Additional Information

**How to cite this article**: Mandic Maravic, V. *et al.* Sex Differences in Autism Spectrum Disorders: Does Sex Moderate the Pathway from Clinical Symptoms to Adaptive Behavior?. *Sci. Rep.* **5**, 10418; doi: 10.1038/srep10418 (2015).

## Figures and Tables

**Table 1a:** Sex-specific sample characteristics (age, diagnosis, clinical characteristics).

**Characteristics**	**Females (N = 25)**	**Males (N = 83)**	**Test statistics**	**Sig.**	**ANCOVA (controlling for age and Vineland-II score)**	
	**Mean (SD) or frequencies**	**Mean (SD) or frequencies**	**t-test / Chi**^**2**^**-test**		**F (sex)**	**Sig.**
Age	6.30 (4.46)	6.86 (4.31)	t = −0.570	0.570		
Diagnosis: Autism/Other ASD	14/11	69/14	Chi^2^ = 7.951	0.005		
ADI-R score A	18.12 (5.34)	19.59 (5.45)	t = 1.187	0.238	0.908	0.343
ADI-R score B	9.20 (3.069)	9.63 (3.11)	t = 0.602	0.548	0.701	0.404
ADI-R score C	4.28 (2.84)	4.51 (2.11)	t = −0.369	0.715	0.015	0.902

ADI-R score A: Social reciprocity domain, current algorithm.

ADI-R score B: Communication domain, current algorithm.

ADI-R score C: Restrictive and repetitive behavior, current algorithm.

ANCOVA: Univariate analysis of covariance between males and females, with age and VINELAND-II score as covariate.

**Table 1b:** Sex-specific sample characteristics (adaptive behavior).

**Characteristics**	**Females (N** = **25)**	**Males (N** = **83)**	**t-test**	**Sig.**	**ANCOVA (controlling for age and ADI-R scores)**	
	**Mean (SD)**	**Mean (SD)**			**F (sex)**	**Sig.**
Vineland-II score	61.40 (14.19)	56.53 (15.05)	−1.436	0.154	1.657	0.201
Vineland – C	53.92 (11.71)	48.67 (12.36)	−1.882	0.063	2.648	0.107
Vineland– DLS	70.80 (18.05)	62.71 (18.94)	−1.892	0.061	3.570	0.062
Vineland – S	57.92 (14.52)	56.73 (12.52)	0.400	0.690	0.098	0.754
Vineland – MS	79.00 (15.51)	73.67 (15.29)	1.312	0.193	0.520	0.473

Vineland-II score: Vineland adaptive behavior scale, composite score.

Vineland – C score: Vineland communication scale.

Vineland – DLS score: Vineland daily living skills scale.

Vineland – S score: Vineland socialization scale.

Vineland – MS score: Vineland motor skills scale.

ANCOVA: Univariate analysis of covariance between males and females, with age and ADI-R scores as covariates.

**Table 2:** Linear regression model of adaptive behavior among males and females, with age and all ADI-R scores as predictors (presenting only significant predictors for each dependant variable).

**Dependent variable**	**Males**			**Females**		
	**Significant predictors (Beta)**	**R**^**2**^	**F**	**Significant predictors (Beta)**	**R**^**2**^	**F**
Vineland-II	Age (–0.723***)	0.636	34.126***	Age (–0.750***)	0.667	10.019**
	ADI-R score A (–0.227*)					
Vineland-C	Age (–0.607***)	0.393	12.611***	Age (–0.630**)	0.585	7.057**
				ADI-R score C (–0.403^¥^)		
Vineland-DLS	Age (–0.704***)	0.614	31.013***	Age (–0.667**)	0.595	7.352**
	ADI-R score A (–0.218*)					
	ADI-R score C (–0.167*)					
Vineland-S	Age (–0.755***)	0.681	41.642***	Age (–0.665***)	0.677	10.488***
	ADI-R score C (–0.214**)			ADI-R score A (0.433^¥^)		
				ADI-R score C (–0.428*)		
Vineland-MS	Age (–0.315*)	0.245	4.229**	Age (–0.640*)	0.407	2.402
	ADI-R score A (–0.370*)					

ADI-R score A: Social reciprocity domain, current algorithm.

ADI-R score B: Communication domain, current algorithm.

ADI-R score C: Restrictive and repetitive behavior, current algorithm.

Vineland-II score: Vineland adaptive behavior scale, composite score.

Vineland – C score: Vineland communication scale.

Vineland – DLS score: Vineland daily living skills scale.

Vineland– S score: Vineland socialization scale.

Vineland– MS score: Vineland motor skills scale.

*** <0.001; ** <0.01; * <0.05; ¥ ≤0.07.

**Table 3:** Significant sex moderation effects in ADI-R predictions of Vineland scores (with age and other two ADI-R scores as covariates).

**Significantly moderated associations (Predictor to outcome)**	**Interaction of sex with ADI-R score A B (LLCI-ULCI)**	**Male conditional effect B (LLCI-ULCI)**	**Female conditional effect B (LLCI-ULCI)**
ADI-R score A to Vineland-DLS score	1.232 (0.138-2.326)*	−0.724 (−1.352 - −0.095)*	0.508 (−0.600 – 1.622)
ADI-R score A to Vineland-S score	1.042 (0.352-1.732)**	−0.197 (−0.593-0.199)	0.845 (0.142 – 1.547)*

ADI-R score A: Social reciprocity domain, current algorithm.

Vineland – DLS score: Vineland daily living skills scale.

Vineland– S score: Vineland socialization scale.

B: unstandardized coefficient.

LLCI / ULCI: lower limit 95% confidence interval / upper limit 95% confidence interval.

** <0.01; * <0.05.
